# Bayesian Dimensionality Assessment for the Multidimensional Nominal Response Model

**DOI:** 10.3389/fpsyg.2017.00961

**Published:** 2017-06-16

**Authors:** Javier Revuelta, Carmen Ximénez

**Affiliations:** Department of Psychology, Autonoma University of MadridMadrid, Spain

**Keywords:** multidimensional nominal response model, multidimensional item response theory, standardized generalized discrepancy measure, WAICC, LOO, Bayesian inference

## Abstract

This article introduces Bayesian estimation and evaluation procedures for the multidimensional nominal response model. The utility of this model is to perform a nominal factor analysis of items that consist of a finite number of unordered response categories. The key aspect of the model, in comparison with traditional factorial model, is that there is a slope for each response category on the latent dimensions, instead of having slopes associated to the items. The extended parameterization of the multidimensional nominal response model requires large samples for estimation. When sample size is of a moderate or small size, some of these parameters may be weakly empirically identifiable and the estimation algorithm may run into difficulties. We propose a Bayesian MCMC inferential algorithm to estimate the parameters and the number of dimensions underlying the multidimensional nominal response model. Two Bayesian approaches to model evaluation were compared: discrepancy statistics (DIC, WAICC, and LOO) that provide an indication of the relative merit of different models, and the standardized generalized discrepancy measure that requires resampling data and is computationally more involved. A simulation study was conducted to compare these two approaches, and the results show that the standardized generalized discrepancy measure can be used to reliably estimate the dimensionality of the model whereas the discrepancy statistics are questionable. The paper also includes an example with real data in the context of learning styles, in which the model is used to conduct an exploratory factor analysis of nominal data.

Nominal variables are routinely obtained from a number of item response formats in the fields of ability measurement, attitude scales, sample surveys, market research, etc. One example is multiple-choice items that contain one correct option and several distractors. When the data come from multiple-choice items, the factorial analysis of nominal variables often proceeds by dichotomizing the data into right and wrong responses and submitting the dichotomous data matrix into a categorical factor analysis procedure. However, there are situations when dichotomization is not an option because the interest is in the relation between latent dimensions and the response categories. For example, in an item from a market research each category may represent a purchase option and there is not a natural way to dichotomize the data.

The factorial analysis of responses that have an implicit ordering has long been discussed in the psychometric literature as well as their estimation and testing procedures (Christoffersson, 1975; Bartholomew, 1980; Reckase, 2009). Such models are based on a normal or logistic function that links observed responses and dimensions by using a vector of slopes. Moreover, a set of intercept parameters determine the distribution of responses across the categories of the item (Mislevy, 1986). The factor analysis of nominal variables is more recent due to the inherent difficulties of the underlying psychometric model. This model is a multidimensional extension of the nominal response model by Bock (1972), which assumes that items load in a single dimension. In the nominal response model the slopes are parameters of the categories instead of parameters of the items. One item that has three response categories and measures two dimensions (say) would have two thresholds and two slopes for the ordinal model, whereas there would be two thresholds and four slopes for the nominal model (one category has no parameters and the other categories have one slope in each dimension).

Applications of constrained versions of the multidimensional nominal response model (MNRM) have been published in the psychometric literature. For example, Hoskens and de Boeck (2001) applied a constrained MNRM to evaluate cognitive components involved in item solving, in this model parameter constraints are imposed to reflect the components measured by the categories. Johnson and Bolt (2010) developed another version of the MNRM aimed at the separation of a general dimension of ability from secondary dimensions that represent the test taking strategy. In this article, the MNRM will be used in its full generality to conduct an exploratory factor analysis of nominal variables. In the exploratory analysis none of the parameters is fixed to a constant value, except when necessary to identify the model. The extended parameterization of the MNRM introduces difficulties related to interpretation of parameters and estimation. Regarding parameter interpretation, several parameterizations have been introduced by Thissen et al. (2010) and Falk and Cai (2016) aimed at obtaining parameters with a clear meaning. This article focuses on the inferential aspects, in particular on the estimation of the number of dimensions.

The estimation problems of the MNRM emerge because the contingency table of the response patterns is typically too sparse due to the large number of response categories that have to be modeled. Maximum likelihood estimates can be obtained using computer programs such as Latent GOLD (Vermunt and Magidson, 2016). However, the maximum-likelihood estimation algorithm may run into difficulties that render high standard errors when sample size is around a few hundred individuals. Typically convergence problems will show up for the parameters of those categories that have a low response frequency, which can appear even when the sample size is relatively high. For example, with a sample of 500 or more individuals, it is not uncommon to find categories with frequencies below 10, which obviously cannot render stable estimates for the many parameters that describe the category. Apart from the estimation difficulties, testing the fit of the nominal model in the frequentist framework is not easy because goodness-of-fit statistics are based on asymptotic arguments that hardly conform to the realistic conditions of model application.

The statistical problems of the nominal model may be addressed by the definition of prior distributions for the parameters and moving the inference to a Bayesian context. Bayesian inference combines the information from the sample with the information in the prior distributions, which stabilizes estimates, alleviates the problems of lack of convergence for some parameters and provides a means for simulating the posterior distribution of model evaluation statistics.

The purpose of this article is to introduce a Bayesian inferential algorithm for the evaluation of the latent dimensionality of the MNRM. The proposed procedure is based on standard Bayesian estimation algorithms by Markov chain Monte Carlo (MCMC) procedures. Bayesian estimation has already been applied to ordinal responses (Kieftenbeld and Natesan, 2012) and multidimensional models (Levy et al., 2009) in the context of item response theory. Bayesian procedures have been successfully applied to testing model fit by simulating the distribution of evaluation statistics (Sinharay et al., 2006). However, the definition of model evaluation statistics for a nominal model is a newer field of research. We have applied two model evaluation statistics that have been recently proposed in a Bayesian statistical context, the widely applicable information criterion (WAIC) and the leave-one-out cross-validation (LOO), based on the information theory (Gelman et al., 2014b), and which, to our knowledge, have not been applied before in a psychometric context. Moreover, the article includes an extension to the nominal case of the standardized generalized dimensionality discrepancy measure (SGDDM) by Levy et al. (2015). The SGDDM was originally proposed for the evaluation of the dichotomous item response model, and subsequently extended to ordinal factorial models. This article shows that the SGDDM provides useful information for dimensionality assessment of the nominal model.

The rest of the article is organized in the following sections. Section Multidimensional Nominal Response Model describes the MNRM, the constraints for parameter identification, and the rotation problem. The MCMC Bayesian estimation algorithm is presented in Section Bayesian Parameter Estimation, Section Bayesian Model Evaluation describes the model evaluation statistics. Section Simulation Study consists of a simulation study that evaluates the Bayesian inferential algorithm in realistic conditions. Section Real Data Analysis contains a real data study in the context of a questionnaire of learning styles whose response categories represent different learning styles, and there is no implicit order among them. Section Final Remarks concludes the article.

## Multidimensional nominal response model

### The multidimensional nominal response model

The MNRM was introduced by Takane and de Leeuw (1987) and McFadden (1974). Recent developments appear in Thissen et al. (2010). Revuelta (2014) describes maximum likelihood estimation algorithms and a structural model for the dimensions. Suppose that an item is scored in *K* nominal categories. Under the MNRM, the probability of category *k*, where, *k* = 1, …., *K*, conditional on a vector of *D* dimensions in the latent space, θ = (θ_1_, …, θ_*d*_, …, θ_*D*_), is given by the logistic function:

(1) $$\begin{array}{l} P_{k}\left(\theta \right)=\frac{\exp\left(z_{k}\right)}{\overset{K}{\underset{k\prime =1}{\sum }}\exp\left(z_{k\prime }\right)}, \end{array}$$

where *z*_*k*_ is the response value of category *k* and is given by a linear function of the dimensions:

(2) $$\begin{array}{l} z_{k}=c_{k}+a_{k1}\theta_{1}+\cdots +a_{kd}\theta_{d}\cdots +a_{kD}\theta_{D}. \end{array}$$

The parameters of the model in Equation (2) are the intercept *c*_*k*_ and the slopes, *a*_*k*1_,…, *a*_*kd*_,…, *a*_*kD*_. The MNRM is usually estimated under the assumption that the mean of the dimensions is zero; then the intercept represents the value of the response value for an individual whose vector of dimensions is equal to the population mean. The slope *a*_*kd*_ represents the relation of the response value *z*_*k*_ with dimension *d*.

The model in Equations (1) and (2) with only one dimension (*D* = 1) is the nominal response model by Bock (1972). Equation (2) follows the classical parameterization of the model introduced by Bock (1972), although there are newer parameterizations that will be commented below.

An item with *K* categories that measures *D* dimensions has *K* intercepts and *K* × *D* slopes, resulting in *K* × (*D*+1) parameters per item. However, not all of these parameters can be estimated because in that case there would be indeterminacy in the model. This is because if a constant is added to all the utilities, the probability given by Equation (1) remains unchanged. Suppose that we define $z_{k}^{*}=z_{k}+T$, where *T* is a constant. The probability given by (1) is the same irrespective of the value of *T*:

(3) $$\begin{array}{l} \frac{\exp\left(z_{k}^{*}\right)}{\overset{K}{\underset{k\prime =1}{\sum }}\exp\left(z_{k\prime }^{*}\right)}=\frac{\exp\left(z_{k}+T\right)}{\overset{K}{\underset{k\prime =1}{\sum }}\exp\left(z_{k}+T\right)}=\frac{\exp\left(T\right)\exp\left(z_{k}\right)}{\exp\left(T\right)\overset{K}{\underset{k\prime =1}{\sum }}\exp\left(z_{k}\right)}\\ =\frac{\exp\left(z_{k}\right)}{\overset{K}{\underset{k\prime =1}{\sum }}\exp\left(z_{k}\right)}. \end{array}$$

The indeterminacy problem is resolved by imposing a constraint on the utilities. Possibly the easiest methods of identification for the parameters of Equation (2) are *simple constraints* and *deviation constraints*.

Simple constraints consist of setting to zero the response value of one of the categories. Simple constraints are useful for those items that have a reference category against which the other categories are compared, for example, a *don't know* category in an attitude scale or the correct category in a multiple-choice item. Suppose that the reference category is *K*. The parameters *c*_*K*_ and *a*_*K*_ are set to 0, which implies that *z*_*K*_ = 0. The utilities of the remaining categories are interpreted relative to *z*_*K*_ using log-odds. In particular, the parameters of category *k* are indicative of the log-odds of categories *k* and *K*:

(4) $$\begin{array}{l} \log\frac{P_{k}\left(\theta \right)}{P_{K}\left(\theta \right)}=c_{k}+a_{k1}\theta_{1}+\cdots +a_{kD}\theta_{D}. \end{array}$$

Deviation constraints consist of setting to zero the sum of the utilities, $\sum_{k=1}^{K}z_{k}=0$. This constraints implies that the sum of parameters across categories is zero:

(5) $$\begin{array}{l} \sum_{k=1}^{K}c_{k}=\sum_{k=1}^{K}a_{k1}=\cdots =\sum_{k=1}^{K}a_{kD}=0. \end{array}$$

Deviation constraints are useful for those items in which it is undesirable to have one category with zero parameters, which are items that do not have a reference category; Section Real Data Analysis below shows one example. Deviation constraints involve trade-offs between parameters because if one parameter increases, the others should decrease so that the sum of the parameters will be constant at zero. These trade-offs introduce technical complications in the estimation algorithm. For these reasons the model is estimated under simple constraints and the estimates are subsequently transformed to deviation constraints if necessary. Suppose that **ε** is a vector of *K* item parameters under simple constraints [**ε** can be either a vector of intercepts, **ε** = (*c*_1_, …, *c*_*K*_), or a vector of slopes in the same dimension, **ε** = (*a*_1*d*_, …, *a*_*Kd*_)]. Parameters can be transformed to deviation constraints by subtracting the mean of the vector:

(6) $$\begin{array}{l} \varepsilon^{\left(deviation\;constrain\right)}=\varepsilon -\frac{\sum_{k=1}^{K}\varepsilon_{k}}{K} \end{array}$$

For example, suppose that an item has the following intercepts under simple constraints: *c*_1_ = 5, *c*_2_ = 4, and *c*_3_ = 0; these parameters indicate that the probability of categories 1 and 2 is higher than the probability of category 3 for an individual whose vector of dimensions is zero. According to Equation (6) the intercepts under deviation constraints are *c*_1_ = 2, *c*_2_ = 1, and *c*_3_ = −3. Although simple and deviation constraints convey the same information regarding the probabilities of the categories, parameter values under simple constraints will vary depending on which category is used as a reference. When the choice of the reference category is arbitrary deviation constraints are preferred.

Both simple and deviation constraints imply that 1 + *D* item parameters are set to a constant value (one intercept and *D* slopes are fixed). Thus, the number of effective item parameters reduces to *K* × (1 + *D*) − (1 + *D*) = (*K* − 1) × (*D* + 1), which is the result of having one intercept and *D* slopes for *K* − 1 categories.

Recent developments of the MNRM have been proposed by Thissen et al. (2010), Falk and Cai (2016) and Thissen and Cai (2017) to facilitate the interpretation of parameters without altering the statistical properties of the model. The idea of these developments is to separate a vector of item slope parameters from the scoring of the categories. In the newer parameterization the slopes no longer represent the categories but the item, as in the traditional factor models, and categories are represented by vectors of scoring parameters that indicate their ordering in relation to the dimension. In particular, the response value of category *k* for the model by Falk and Cai (2016) is:

(7) $$\begin{array}{l} z_{k}=c_{k}+s_{k1}a_{1}\theta_{1}+\cdots +s_{kd}a_{d}\theta_{d}+\cdots +s_{kD}a_{D}\theta_{D}. \end{array}$$

The parameters of Equation (7) are the intercept, *c*_*k*_, a vector of item slopes, *a*_1_, …, *a*_*d*_, …, *a*_*D*_ and the scoring parameters of category *k* in the *D* dimensions: *s*_*k*1_, ⋯ , *s*_*kd*_, ⋯ , *s*_*kD*_. The intercept has the same interpretation as in Equation (2) and the constraint *c*_1_ = 0 is imposed for identification. The scoring parameter *s*_*kd*_ represents the weight of category *k* in dimension θ_*d*_, and the slope *a*_*d*_ is the weight of the item in θ_*d*_.

The model in Equation (7) assumes that there exists an ordering among the categories albeit unknown. The ordering is represented by the scoring parameters and is estimated from the data. Consider the scoring parameters of the *K* categories in the same dimension θ_*d*_, that *s*_1*d*_, …, *s*_*kd*_, …*s*_*Kd*_. These scores are used to obtain an ordering of the categories according to their weight in the dimension θ_*d*_. The scoring parameters of two categories in θ_*d*_ must be fixed to constant values to identify the model and serve as anchor points. Typically the scores of the first and the last category are fixed as *s*_1*d*_ = 0 and *s*_*Kd*_ = *K* − 1, whereas the values of *s*_*kd*_ for the remaining categories are estimated.

The model in Equation (7) has *D* item slopes, (*K*−2) × *D* scoring parameters and (*K* − 1) estimated intercepts, which renders a total of (*K* − 1) × (*D*+1) estimated parameters. Because the models given in Equations (2) and (7) have the same number of parameters, they are statistically equivalent and cannot be distinguished on the basis of goodness of fit statistics. The choice of parameterization depends on the intended use of the model and interpretation.

The slopes in Equation (2) and the slopes in Equation (7) are related by the equation:

(8) $$\begin{array}{l} a_{kd}=s_{kd}a_{d} \end{array}$$

where *k* = 1, …, *K* and *d* = 1, …, *D*. Suppose that the anchor points for the scoring parameters are *s*_1*d*_ = 0 and *s*_*Kd*_ = *K* − 1. Then, developing Equation (8), item slopes and scoring parameters can be computed from the slopes under simple constraints by the equations:

(9) $$\begin{array}{l} a_{d}=\frac{a_{Kd}}{K-1}\\ s_{kd}=\frac{a_{kd}}{a_{d}}\text{ for }k=2,\ldots ,K-1 \end{array}$$

Note that the intercept parameter is the same in Equations (2) and (7) and there is no need to transform one another.

The model in Equation (7) has been applied to multiple-choice items, in which category *K* is the correct response and categories 1, …, *K* − 1 are distractors. All distractors are wrong but have differential values of correctness that can be estimated from observed responses. In such a case, *s*_1*d*_ = 0 is arbitrarily assigned to the first distractor that serves as a reference, *s*_*Kd*_ = *K* − 1 is the scoring of the correct response, and *s*_*kd*_ is estimated for distractors 2, …, *K* − 1 and represent their degree of correctness. When the estimated value of *s*_*kd*_ is smaller than 0, the interpretation is that distractor *k* is less correct than the first distractor. When the estimated *s*_*kd*_ is higher than *K* − 1, the interpretation is that the item content has to be revised because no distractor should be more correct than the correct category. Apart from the multiple-choice case, the model in Equation (7) is useful for the analysis of ordinal items when the distance between the scores of the categories varies from one pair of categories to another; examples of the application to Likert-type items are given in Falk and Cai (2016).

The classic parameterization of the MNRM is appropriate when the interest is to estimate the relation of each category with each dimension. On the other hand, the parameterization in Equation (7) would be preferable when the interpretation of item slopes and the ordering of the categories are meaningful. For instance, consider the following item taken from a sample survey about social attitudes.

The item in Table 1 is intended to measure traits such as conservatism and religious feelings. The item has a very short stem and almost all the content is contained in the alternatives. For an item like this, the classic parameterization in Equation (2) would suffice because the relevant information is the strength of the association of each category with each dimension. Moreover, in this item there is not a natural choice of the two categories that serve as anchor points for the scoring parameters, and the estimation of slopes associated with the item instead of the categories would not enhance the interpretation of results.

**Table 1:** Example of item from a survey of social attitudes.

Choose the most important attitude that children must learn at home
- Independence
- Hard work
- Responsibility
- Imagination
- Tolerance and respect for other persons
- Perseverance
- Religious faith
- Abnegation
- Obedience
- Don't know

The focus of this paper is on the use of Bayesian methods to estimate the number of dimensions under the MNRM. From a computational point of view, simple constraints are preferable for simplicity and numerical stability. However, the other parameterizations shall be preferred in application depending on the specific items that are being analyzed. The results of this paper regarding Bayesian methods are irrespective of the parameterization and will be equally applicable when using deviation constraints or item slopes and scoring parameters to interpret results. The recommended computational strategy is to estimate the model under simple constraints and transform the output of the estimation algorithm to the other parameterizations if desired.

### Rotation of slopes

Akin to any other factor model, the parameters for the MNRM are subject to rotational indeterminacy. To fix rotation during estimation, we have implemented the same solution as in the NOHARM computer program, which estimates the normal ogive model for dichotomous data (Fraser and McDonald, 1988). The solution consists of setting to zero the first (*t* − 1) slopes for dimensions *t* = 2, …, *D* during estimation. Moreover, the *t*-th slope for dimension *t* is set to 1 to fix the scale of the dimensions, as will be commented in Section Bayesian Parameter Estimation.

Let **A** be the matrix of slopes under simple constraints. The elements of **A** are the slopes for all items and all categories but category *K* (the slopes of category *K* are structural zeros and are not included in **A**). Every item has *K* − 1 vectors of slopes after taking into account that the reference category has no slopes. Then **A** has items ×(*K* − 1) rows and *D* columns. Suppose, for example that a test contains three items with three categories that measure three dimensions. Then, matrix **A** is given by:

(10) $$\begin{array}{l} \text{A}=\left(\begin{array}{ccc} 1 & 0 & 0\\ a_{121} & 1 & 0\\ a_{211} & a_{212} & 1\\ a_{221} & a_{222} & a_{223}\\ a_{311} & a_{312} & a_{313}\\ a_{321} & a_{322} & a_{323} \end{array}\right), \end{array}$$

where the subscripts refer to item, category, and dimension, respectively. For example, *a*_321_ is the slope of item 3 and category 2 in dimension 1. Equation (10) shows the pattern of zeros and ones that have to be imposed on the slopes to avoid rotational indeterminacy during estimation. Bayesian estimation algorithms are applied assuming that these zeros and ones are constant values, and the remaining slopes are estimated. After estimation is complete, the resulting matrix **A** can be rotated to obtain a more interpretable solution.

The vector of utilities can be written in matrix form as:

(11) $$\begin{array}{l} \text{z}=\text{c}+\text{A}\theta . \end{array}$$

Rotation consists of finding a nonsingular rotation matrix **M** and transforming the estimated slopes to rotated slopes, A^*^, by the equation (Lawley and Maxwell, 1971):

(12) $$\begin{array}{l} {\text{A}}^{*}=\text{AM}. \end{array}$$

Rotated scores, θ^*^, are given by:

(13) $$\begin{array}{l} \theta^{*}={\text{M}}^{-1}\theta . \end{array}$$

Because **M** is nonsingular **MM**^-1^ = **I**, where **I** is an identity matrix. Then, rotation does not alter the utilities of the categories because A^*^θ^*^ = **AMM**^−1^**θ** = **Aθ**. Moreover, rotation preserves the identification constraints. The unrotated and rotated slopes have the same type of identification constraints, either simple or deviation constraints.

Matrix **M** can be obtained by any of the algorithmic methods that are common in factor analysis for orthogonal or oblique rotation: varimax, oblimin, etc. General purpose computer algebra systems or statistical languages such as R (R Development Core Team, 2011) have functions that receive a matrix **A**, generate **M** and perform rotation according to the desired criterion.

### Estimation of the model

The process of estimating the model consists of three steps:

1. Apply the Bayesian estimation algorithm described in Section Bayesian Parameter Estimation to estimate the model under simple constraints and imposing the pattern of zeros and ones described in Section Rotation of Slopes to avoid rotational indeterminacy. Model evaluation statistics described in Section Bayesian Model Evaluation are used to test model fit. If the model does not fit, a model with a higher number of dimensions has to be estimated. The output of this step is a model parameterized with simple constrains that satisfactory fits the data.
2. Estimated parameters may be transformed to deviation constraints with Equation (6) or to the item slopes and scoring parameters with Equation (9). The transformation of parameterizations is optional and depends on the intended interpretation and the type of items.
3. Rotate the slopes using a rotation algorithm or by graphical rotation. This step is optional. The choice of a rotation method depends on the judgment of the data analyst.

## Bayesian parameter estimation

The MNRM has a heavy parameterization because there are slopes for (*K* − 1) item categories. Inference is facilitated by incorporating additional information through prior distributions, which contribute to obtain stable inferences. More specifically, item parameters are estimated via Markov chain Monte Carlo (MCMC; Gelman et al., 2014a). The application of Bayesian MCMC simulation to item response modeling is originally due to Albert (1992), Albert and Chib (1993) and Patz and Junker (1999a,b). An introduction to the topic is given by Baker and Kim (2004), and a book-length treatment can be found in Fox (2010).

MCMC provides draws from the posterior distribution of item parameters. These samples can be summarized using descriptive statistics to obtain a point-estimate, the simulated expected a-posteriori estimate (EAP), and the posterior variance. Previous application of MCMC to factorial and multidimensional item response models can be seen, for example, in Béguin and Glas (2001), Edwards (2010) and Chen (2016).

One property of factorial models is that the orientation of the dimensions can be reverse without altering the fit of the model. That is, if one dimension θ_*d*_ and the slopes in that dimension are multiplied by −1, the resulting model will be statistically equivalent. This problem is especially compelling for MCMC estimation because several Markov chains of simulated parameters are run in parallel and some procedure must be applied to ensure that all chains are oriented in the same direction. In this article we have fixed the orientation of the dimension trait by setting the first slope of each dimension trait to 1, as mentioned in Section Rotation of Slopes. This is compensated by freeing the standard deviations of the dimensions (σ_1_, …, σ_*d*_, …, σ_*D*_), for the total number of estimated parameters to remain unchanged.

The estimated parameters are the intercepts (***c***), the slopes (***a***), and the standard deviations (σ), whereas **θ** is regarded as a random effect. Let *i* = 1, …, *N* be the number of the examinee and *j* = 1, …, *J* be the item number, the following prior distributions are used:

(14) $$\begin{array}{l} \;c_{jk}\sim \text{normal}(0,\delta )\\ \;a_{jkd}\sim \text{normal}(0,\gamma )\\ \sigma_{d}\sim \text{lognormal}(\mu ,\tau )\\ \;\theta_{id}\sim \text{normal}(0,\sigma_{d}) \end{array}$$

The hyper-parameters δ, γ, μ, and τ will be held to constant values in this article. A more general procedure has been proposed by Natesan et al. (2016), in which the hyper-parameters are estimated to avoid bias. MCMC simulation is run using the Stan computer language (Gelman et al., 2015). Stan is based on a Hamiltonian dynamics sampling algorithm that supersedes the traditional Gibbs-sampling used in MCMC and converges to the posterior distribution with chains of shorter length (Martín-Fernández and Revuelta, 2017). Convergence of parameter estimates is monitored by the scale reduction factor, $\sqrt{R}$ statistic, in Gilks et al. (1996) and Brooks and Gelman (1998).

## Bayesian model evaluation

### Model evaluation via posterior predictive checks

A crucial problem when performing an exploratory factor analysis is the selection of the number of dimensions. In the frequentist framework, there are many criteria suitable for this purpose, the chi-square goodness of fit statistics, the RMSEA statistic for the hypothesis of close fit, parallel analysis, and many others (Brown, 2006). However, these quantities are not immediately transferable to the Bayesian context, where model fit is typically tested by computationally intensive resampling methods that simulate the posterior predictive distribution of evaluation statistics (Gelman et al., 2014a).

One readily interpretable model evaluation statistic is the standardized generalized dimensionality discrepancy measure (SGDDM), introduced by Levy et al. (2015) as a variant of the procedure in Levy et al. (2009). The SGDDM is a quantification of the standardized model-based covariance between two items, *j* and *j*′, and thus, it is interpretable as a model-based posterior correlation between a pair of responses.

The SGDDM applies to dichotomous and ordinal responses (Yel et al., 2013). In this article, we generalize the SGDDM to the nominal case and compute the covariance between pairs of categories. The response of individual *i* to item *j* is represented by a vector of *K* − 1 binary variables. The variable *X*_*ijk*_ takes the value 1 when the response is *k* and 0 otherwise; thus, the upper category *K* is represented by a vector of zeros. The SGDDM for the pairing of categories *k* and *k*′ of items *j* and *j*′ is given by:

(15) $$\begin{array}{l} SGDDM_{jk,j\prime k\prime }=\frac{\frac{1}{N}|\overset{N}{\underset{i=1}{\sum }}\left(X_{ijk}-P_{ijk}\right)\left(X_{ij\prime k\prime }-P_{ij\prime k\prime }\right)|}{\sqrt{\frac{1}{N}\overset{N}{\underset{i=1}{\sum }}{\left(X_{ijk}-P_{ijk}\right)}^{2}}\sqrt{\frac{1}{N}\overset{N}{\underset{i=1}{\sum }}{\left(X_{ij\prime k\prime }-P_{ij\prime k\prime }\right)}^{2}}}, \end{array}$$

where *N* is the number of individuals, *J* is the number of items, and *P*_*ijk*_ is the response function given by Equation (1). *P*_*ijk*_ is computed conditional on the item parameters and the values of **θ** realized in the MCMC simulation.

Posterior predictive checks proceed as follows. Suppose that ω_1_, …, ω_*l*_, …, ω_*L*_ are vectors of parameters simulated in the MCMC chains, that is **ω**_*l*_ = (***c***, ***a***, ***σ***, ***θ***). Conditional on ω_*l*_, simulate a matrix of predicted responses, ${\text{X}}_{l}^{pred.}$, of the same size as the observed response matrix; compute the value of the $SGDDM_{jk,j\prime k\prime }$ for the observed and predicted responses, denoted by $SGDDM_{jk,j\prime k\prime }\left(\text{X};\omega_{l}\right)$ and $SGDDM_{jk,j\prime k\prime }\left({\text{X}}^{pred};\omega_{l}\right)$. A posterior predictive *p*-value for the paring of categories (*jk*) and (*j*′*k*′) is given by:

(16) $$\begin{array}{l} p_{post_{jk,j\prime k\prime }}=\frac{1}{L}\overset{L}{\underset{l=1}{\sum }}\delta \left(\begin{array}{c} SGDDM_{jk,j\prime k\prime }\left({\text{X}}^{pred};\omega_{l}\right)\\ \geq SGDDM_{jk,j\prime k\prime }\left(\text{X};\omega_{l}\right) \end{array}\right), \end{array}$$

where δ(·) returns the value 0 or 1 when its argument is false or true, respectively.

A discrepancy statistic for the whole model is obtaining by averaging the value of $SGDDM_{jk,j\prime k\prime }$ for all nonredundant pairs of items and categories:

(17) $$\begin{array}{l} SGDDM\left(\text{X};\omega \right)=\frac{2}{J\left(K-1\right)\left(J\left(K-1\right)-1\right)}\\ \overset{J}{\underset{j=1}{\sum }}\overset{J}{\underset{j\prime =j+1}{\sum }}\overset{K-1}{\underset{k=1}{\sum }}\overset{K-1}{\underset{k\prime =1}{\sum }}SGDDM_{jk,j\prime k\prime }. \end{array}$$

The posterior predictive *p*-value, *p*_*post*_, is the proportion of cases in which the *SGDDM* for the predicted data is equal to or higher than the *SGDDM* for the observed data; that is:

(18) $$\begin{array}{l} p_{post}=\frac{1}{L}\overset{L}{\underset{l=1}{\sum }}\delta \left(SGDDM\left({\text{X}}^{pred};\omega_{l}\right)\geq SGDDM\left(\text{X};\omega_{l}\right)\right), \end{array}$$

Alternatively, Levy and Svetina (2011) recommend the evaluation of model adequacy by plotting the values of *SGDDM*(**X**; **ω**) and *SGDDM*(**X**^*pred*^; **ω**) to evaluate the magnitude of the discrepancies between the two vectors instead of computing a posterior predictive *p*-value that loses the information of the magnitude of the difference between *SGDDM*(**X**; **ω**) and *SGDDM*(**X**^*pred*^; **ω**).

### Model selection using discrepancy statistics

Model evaluation by posterior predictive checks is a computationally intensive method based on resampling data. Several summary statistics have been proposed to avoid resampling. Possibly, the most popular statistic within the Bayesian context is the deviance information criterion (DIC; Spiegelhalter et al., 2002). DIC is a version of the Akaike information criterion (AIC) that combines the log posterior probability of the model with an estimation of the effective number of parameters.

Recently, several alternatives to DIC have been proposed in the area of Bayesian inference to overcome the dependence of the DIC on a precise point-wise estimator and its assumption of posterior normality. These new statistics are the widely applicable information criteria (WAIC; Watanabe, 2010, 2013) and the leave-one-out cross validation (LOO, Gelman et al., 2014b). The WAIC and the LOO are approximations to cross-validation computed from a single matrix of observed data. All of these measures are based on adjusting the log predictive density of the observed data by subtracting an approximate bias correction based on the effective number of estimated parameters.

Similar to AIC and other measures of model adequacy based on information theory, WAIC and LOO quantify the discrepancy between the model and the data that also take into account model complexity. The purpose is not to test a hypothesis of model fit but to compare several competing models and select the one that most closely approaches the data. The WAIC closely approximates cross-validation although it is computed in a single sample instead of re-fitting the model using different samples. The WAIC is potentially useful in the psychometric context because it still works with highly parameterized models, where other alternatives such as AIC and DIC are no longer applicable. However, to our knowledge, they have not been previously applied to item response or factorial models.

## Simulation study

A Monte Carlo simulation study was conducted to evaluate the performance of the SGDDM and the discrepancy measures (DIC, WAIC, and LOO) in recovering the true number of dimensions for the MNRM.

### Simulation conditions and analysis

We simulated 50 data sets from models with one, two, and three dimensions. Models with one, two, and three dimensions are estimated from each simulated sample. We have used only a limited number of samples because MCMC is highly time consuming and the simulation study has to be kept within our limit of computational resources. The figure of 50 samples was taken from Levy et al. (2015), who ran similar simulations and pointed out that this figure is sufficient to identify broad patterns in the data.

Two set of prior distributions were used, informative priors and uniform priors. Informative priors are given in Equation (14), the values of δ and γ set to 3 because, in our previous experience, this value renders a relatively flat prior that at the same time avoids the occurrence of extreme values in the estimated parameters. The prior distribution for σ_*d*_ was more stringent to avoid excessive indeterminacy in the scale of the dimension. σ_*d*_ had a lognormal (0, 0.5) prior, which has a median of 1, an expectation of 1.13, and a standard deviation of 0.6. This lognormal prior is the same as the one used by the BILOG computer software (Zimowski et al., 1996). It is preferable to set the median of the lognormal to 1 instead of to the expected value because the lognormal distribution has a thick right tail and a significant skewness, and high values of σ_*d*_ are realized in the simulated samples if the distribution is too flat. Thus, the informative priors are:

(19) $$\begin{array}{l} c_{jk}\sim \text{normal}\left(0,\;3\right)\\ a_{jkd}\sim \text{normal}\left(0,\;3\right)\\ \sigma_{d}\sim \text{lognormal}\left(0,\;0.5\right)\\ \theta_{id}\sim \text{normal}\left(0,\;\sigma_{d}\right) \end{array}$$

And the uniform priors are:

(20) $$\begin{array}{ll} c_{jk}\sim \text{uniform}\left(-10,\text{10}\right)\\ a_{jkd}\sim \text{uniform}\left(-10,\text{10}\right)\\ \sigma_{d}\sim \text{lognormal}\left(0,\;0.5\right) & \theta_{id}\sim \text{normal}\left(0,\;\sigma_{d}\right) \end{array}$$

The simulation was repeated with sample sizes of 250, 500, and 1,000 simulees for each number of dimensions. The total number of conditions is 18 (3 values of dimensions × 3 sample sizes × 2 sets of priors). Responses were simulated from a test with 4 items with four categories each one.

Data were simulated using R version 3.2.5. (R Development Core Team, 2011) and fitted in Stan version 2.9.0–3 (Stan Development Team, 2016). We used four Markov chains of 2,000 samples each one, the first 1,000 samples constitute a start-up period and are discarded, and estimation is based on the 4,000 samples of parameters obtained from merging the second half of the chains. These figures are the default values for the Stan program.

Deviance measures, DIC, WAIC, and LOO were computed using the loo R package (Vehtari et al., 2016). Moreover, a sample of predicted responses was generated for each sample of simulated parameters to compute the posterior predictive value of the SGDDM. For each condition, potential scale reduction factor, indicated by $\sqrt{R}$, was computed to evaluate convergence of the chains (Brooks and Gelman, 1998). The true parameters for the simulation appear in Table 2; true values of **θ** were generated from a standard normal distribution and dimensions are uncorrelated.

**Table 2:** Item parameters used in data generation.

**Item**	**Category**	***c***	***a*_1_**	***a*_2_**	***a*_3_**
1	1	−1	1.0	0.0	0.0
	2	0	0.5	1.0	0.0
	3	1	−1.0	0.5	1.0
	4	0	0.0	0.0	0.0
2	1	−1	1.0	−0.5	−1.0
	2	0	0.5	1.0	−0.5
	3	1	−1.0	0.5	0.5
	4	0	0.0	0.0	0.0
3	1	−1	1.0	0.5	0.5
	2	0	0.5	−0.5	−1.0
	3	1	−1.0	1.0	−0.5
	4	0	0.0	0.0	0.0
4	1	−1	1.0	1.0	−0.5
	2	0	0.5	0.5	0.5
	3	1	−1.0	−0.5	−1.0
	4	0	0.0	0.0	0.0

*Category 4 is the reference category and its parameters have been set to zero to identify the model. Column a_1_ is used for the model with one dimension, columns a_1_ and a_2_ are used for the model with two dimensions, and columns a_1_ to a_3_ are used for the model with three dimensions*.

The analysis of simulation results includes the means of the model evaluation statistics, the empirical proportion of rejections (EPR) of the estimated model, the empirical proportion of selection (EPS) and the root mean square errors (RMSE) of estimated parameters. The EPR applies to the SGDDM only. The SGDDM can be used to test the null hypothesis that a model fits using *p*_*post*_ as the *p*-value of the test. The null hypothesis is rejected when *p*_*post*_ ≤ 0.05. The proportion of simulated samples in which the model is rejected is the EPS. When the model in the null hypothesis (that is, the model used to compute the SGDDM) is the same as the model used to simulate the samples, the EPR is an estimate of the Type I error rate of the SGDDM. When the model in the null hypothesis does not coincide with the model used to simulate the data, the SGDDM is an estimate of the statistical power of the test.

The EPS applies to the model discrepancy statistics, DIC, WAIC, and LOO. In contrast to the SGDDM, the discrepancy statistics are not used to test a hypothesis but to select the best model from a number of competing models. Recall that three models (with one, two, and three dimensions) are estimated from each simulated sample. The discrepancy statistic evaluates the distance between the model and the data, and the model that minimizes the discrepancy statistic is selected. The EPS of a model is the proportion of times that a model is selected in the 50 simulated samples.

The RMSE measures the difference between the true and the estimated parameters to evaluate parameter recovery (Natesan et al., 2016). Although the main purpose of the present simulation is the recovery of the number of dimensions instead of the recovery of parameters, the RMSE will be used to compare the estimation provided by the informative and uniform priors.

### Results and discussion

Table 3 contains the results of model evaluation when the generating model is one-dimensional, and models with one, two, and three dimensions are estimated and with informative priors. The results differ from one statistic to another. The SGDDM never rejects the one-dimension model (a model is retained when *p*_*post*_ > 0.05); models with two and three dimensions are also retained by the SGDDM, as they are generalizations of the one-dimension model. The DIC consistently supports the one-dimension model; however, WAIC and LOO showed a tendency to over-factor and supported the model with three dimensions.

**Table 3:** Model evaluation statistics for the simulation study.

		**SGDDM**				**DIC**		**WAIC**		**LOO**	
***N***	**Est**.	**M-Obs**.	**M-Sim**.	**M-*p_*pos*_*_*t*_**	**EPR**	**Mean**	**EPS**	**Mean**	**EPS**	**Mean**	**EPS**
250	1D	0.072	0.070	0.42	0.00	2,542.0	10.00	2,295.7	0.00	2,312.2	0.22
	2D	0.069	0.070	0.55	0.00	3,079.5	0.00	2,281.7	0.24	2,305.9	0.58
	3D	0.068	0.070	0.59	0.00	3,586.0	0.00	2,277.9	0.76	2,305.7	0.20
500	1D	0.058	0.058	0.47	0.00	5,023.3	10.00	4,594.5	0.00	4,621.4	0.16
	2D	0.057	0.058	0.57	0.00	6,101.4	0.00	4,571.7	0.20	4,608.8	0.64
	3D	0.056	0.057	0.59	0.00	7,227.7	0.00	4,565.3	0.80	4,611.8	0.20
1,000	1D	0.049	0.048	0.44	0.00	10,047.3	10.00	9,099.0	0.00	9,142.3	0.18
	2D	0.047	0.048	0.58	0.00	12,103.0	0.00	9,066.0	0.04	9,122.5	0.46
	3D	0.047	0.048	0.61	0.00	14,537.9	0.00	9,052.0	0.96	9,124.5	0.36

*Generating model D = 1 and informative priors*.

*Est. is the estimated model. M-Obs. is the mean of the observed SGDDM. M-Sim. is the mean of the simulated SGDDM. M-p_post_ is the mean of the p_post_. EPR is the empirical proportion of rejection multiplied by 100, where a model is rejected when p_post_ ≤ 0.05. EPS is the empirical proportion of selection*.

The results for the conditions with a two-dimensional generating model and informative priors appear in Table 4. In these conditions, it would be desirable to reject the one- and three-dimensional models. SGDDM in general rejects the one-dimension model and retains models with two and three dimensions. Discrepancy measures were used for model selection; for each simulated sample, the model that minimizes the discrepancy measure is the one selected. The discrepancy measures exhibit disparate results; DIC consistently supports the one-dimension model, WAIC had a preference for the three-dimension model, whereas LOO discards the one-dimension model and distributes preferences between the two- and the three-dimension model.

**Table 4:** Model evaluation statistics for the simulation study.

		**SGDDM**				**DIC**		**WAIC**		**LOO**	
***N***	**Est**.	**M-Obs**.	**M-Sim**.	**M-*p_*pos*_*_*t*_**	**EPR**	**Mean**	**EPS**	**Mean**	**EPS**	**Mean**	**EPS**
250	1D	0.089	0.070	0.02	0.86	2,534.9	10.00	2,285.6	0.00	2,304.5	0.00
	2D	0.069	0.069	0.47	0.00	2,980.9	0.00	2,199.9	0.12	2,199.9	0.50
	3D	0.068	0.068	0.54	0.00	3,755.7	0.00	2,187.3	0.88	2,187.3	0.50
500	1D	0.079	0.058	0.00	0.98	5,114.2	10.00	4,594.7	0.00	4,623.5	0.00
	2D	0.057	0.056	0.42	0.00	6,027.7	0.00	4,464.8	0.04	4,527.8	0.64
	3D	0.056	0.056	0.52	0.00	7,593.6	0.00	4,451.4	0.96	4,529.6	0.36
1,000	1D	0.078	0.049	0.00	10.00	10,204.2	10.00	9,089.4	0.00	9,136.8	0.00
	2D	0.048	0.047	0.40	0.00	12,013.9	0.00	8,780.5	0.02	8,907.7	0.54
	3D	0.047	0.047	0.49	0.00	15,398.4	0.00	8,757.6	0.98	8,906.8	0.46

*Generating model D = 2 and informative priors*.

*Est. is the estimated model. M-Obs. is the mean of the observed SGDDM. M-Sim. is the mean of the simulated SGDDM. M-p_post_ is the mean of the p_post_. EPR is the empirical proportion of rejection multiplied by 100, where a model is rejected when p_post_ ≤ 0.05. EPS is the empirical proportion of selection*.

Table 5 contains the results for the generating model with three dimensions and informative priors. The SGDDM clearly rejects the one-dimension model, and the two-dimension model is rejected or not depending on sample size. The DIC always supports the one-dimension model, the WAIC always selects the three-dimension model and the LOO shows a preference for the three-dimension model but it is a little bit more conservative than the WAIC, and the two-dimension model has a nonzero proportion of selection.

**Table 5:** Model evaluation statistics for the simulation study.

		**SGDDM**				**DIC**		**WAIC**		**LOO**	
***N***	**Est**.	**M-Obs**.	**M-Sim**.	**M-*p_*pos*_*_*t*_**	**EPR**	**Mean**	**EPS**	**Mean**	**EPS**	**Mean**	**EPS**
250	1D	0.093	0.072	0.01	0.92	2,564.9	10.00	2,272.3	0.00	2,294.0	0.00
	2D	0.078	0.070	0.19	0.12	3,238.9	0.00	2,222.2	0.00	2,262.0	0.10
	3D	0.070	0.069	0.46	0.00	4,315.8	0.00	2,188.1	10.00	2,245.0	0.90
500	1D	0.099	0.059	0.00	10.00	5,308.4	10.00	4,726.4	0.00	4,760.0	0.00
	2D	0.066	0.057	0.05	0.66	6,267.0	0.00	4,585.6	0.00	4,653.5	0.08
	3D	0.057	0.058	0.06	0.00	8,621.4	0.00	4,519.1	10.00	4,626.8	0.92
1,000	1D	0.082	0.050	0.00	10.00	10,598.8	10.00	9,337.7	0.00	9,403.6	0.00
	2D	0.056	0.046	0.02	0.86	13,185.2	0.00	9,070.7	0.00	9,197.4	0.00
	3D	0.064	0.046	0.45	0.00	18,358.9	0.00	8,925.7	10.00	9,133.6	10.00

*Generating model D = 3 and informative priors. Est. is the estimated model. M-Obs. is the mean of the observed SGDDM. M-Sim. is the mean of the simulated SGDDM. M-p_post_ is the mean of the p_post_. EPR is the empirical proportion of rejection multiplied by 100, where a model is rejected when p_post_ ≤ 0.05. EPS is the empirical proportion of selection*.

**Figure 1:**
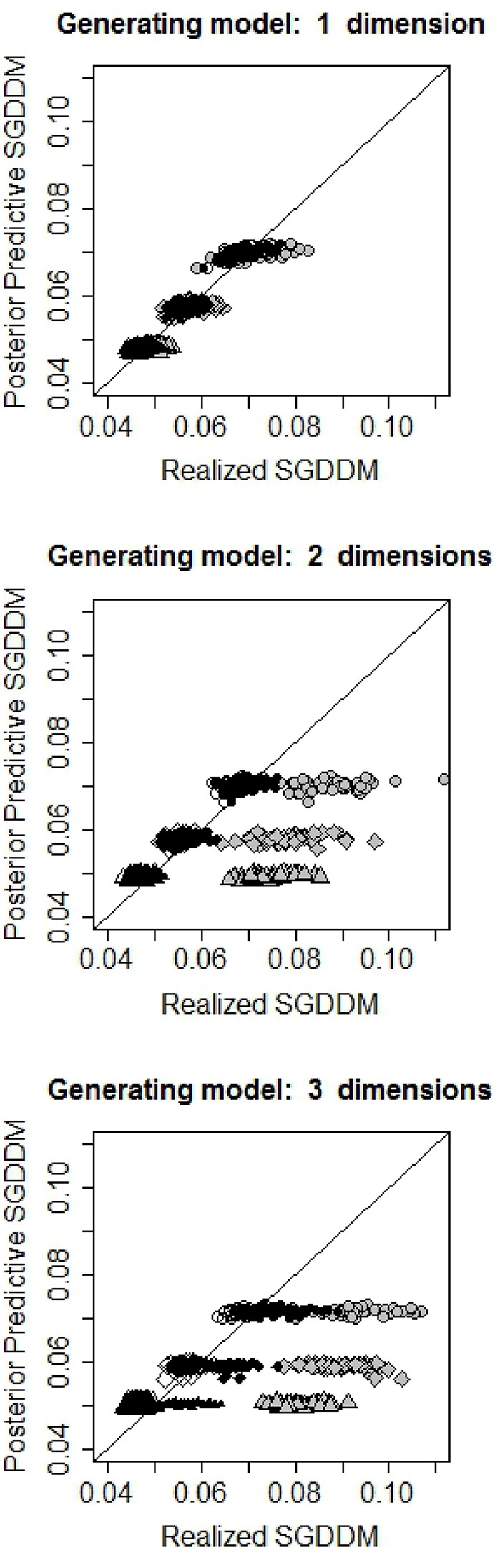
Scatterplot of the realized and predicted values of SGDDM. The line indicates equality of realized and predicted values and is included as a reference. Gray, black and white symbols refer to fitted models with one, two, and three dimensions, respectively. Circles, rhombs and triangles stand for 250, 500, and 1,000 simulees, respectively.

Figure 1 contains the scatter plot of the realized and posterior predictive values of the SGDDM for each condition. The interest of this figure is to appreciate how simulated and realized values overlap when the estimated model has the same or a larger number of dimensions than the simulating model. When the estimated model is under-dimensions, the realized SGDDM is higher than the posterior predictive one and the points are moved to the right of the figure.

The results were almost the same when using uniform priors. For example, Table 6 contains the results for the generating model with two dimensions and uniform priors. The comparison of this table with Table 4 shows that the only difference is that DIC has a stronger tendency of over-fitting when using uniform priors. The results for the other conditions with uniform priors will not be repeated here for brevity.

**Table 6:** Model evaluation statistics for the simulation study.

		**SGDDM**				**DIC**		**WAIC**		**LOO**	
***N***	**Est**.	**M-Obs**.	**M-Sim**.	**M-*p_*pos*_*_*t*_**	**EPR**	**Mean**	**EPS**	**Mean**	**EPS**	**Mean**	**EPS**
250	1D	0.091	0.071	0.01	10.00	2,478.4	10.00	2,279.5	0.00	2,301.4	0.00
	2D	0.069	0.068	0.43	0.00	3,624.4	0.00	2,150.1	0.00	2,230.8	0.02
	3D	0.069	0.067	0.40	0.00	6,187.2	0.00	2,053.4	10.00	2,204.0	0.98
500	1D	0.077	0.058	0.00	0.98	5,004.4	10.00	4,588.4	0.00	4,619.1	0.00
	2D	0.057	0.055	0.40	0.00	9,812.9	0.00	4,372.8	0.00	4,499.0	0.02
	3D	0.056	0.054	0.37	0.00	19,616.7	0.00	4,204.0	10.00	4,446.6	0.98
1,000	1D	0.081	0.050	0.00	10.00	9,888.4	10.00	9,079.6	0.00	9,128.1	0.00
	2D	0.056	0.045	0.32	0.00	26,458.3	0.00	8,542.0	0.02	8,820.6	0.06
	3D	0.047	0.045	0.31	0.00	65,564.5	0.00	8,142.4	0.98	8,674.7	0.94

Generating model D = 2 and uniform priors

*Est. is the estimated model. M-Obs. is the mean of the observed SGDDM. M-Sim. is the mean of the simulated SGDDM. M-p_post_ is the mean of the p_post_. EPR is the empirical proportion of rejection multiplied by 100, where a model is rejected when p_post_ ≤ 0.05. EPS is the empirical proportion of selection*.

The results about recovery of parameters are summarized in Table 7, which contains the averages of the root mean squared error (RMSE) between true and estimated parameters. Recall that one important motivation for moving the inference to the Bayesian context is the complex parameterization of the model and the uncertainty associated with parameter estimates. The conditions with uniform prior essentially provide a maximum-likelihood estimate bounded within the limits of the support of the prior distribution. As expected, the imprecision of the estimates with uniform priors increases with the number of dimensions because more dimensions imply more estimated parameters. The informative prior rendered smaller RMSEs than the uniform prior, and this effect is more prominent as the number of dimensions increases. The standard deviation of the dimension is the parameter that is less affected by the choice of prior distributions. Regarding slopes and intercept, the results show that informative priors stabilize estimates when the sample is not large and the model contains two or more dimensions.

**Table 7:** Average of the RMSE for the estimated parameters.

		**Informative priors**			**Uniform priors**		
***N***	**Est**.	**Params-*c***	**Params-*a***	**Params-σ**	**Params-*c***	**Params-*a***	**Params-σ**
250	1D	0.489	0.377	0.499	0.514	0.406	0.748
	2D	0.590	0.559	0.416	0.715	0.738	0.642
	3D	0.614	0.559	0.428	1.001	1.007	0.573
500	1D	0.419	0.311	0.489	0.430	0.325	0.761
	2D	0.475	0.377	0.462	0.574	0.546	0.675
	3D	0.511	0.455	0.456	0.840	0.924	0.595
1,000	1D	0.363	0.294	0.409	0.387	0.511	0.653
	2D	0.445	0.407	0.354	0.607	0.746	0.535
	3D	0.458	0.437	0.423	0.770	0.924	0.562

*RMSE is the root mean square error. The number of dimensions in the simulating model is the same as in the estimated model*.

In conclusion, the SGDDM has proven to be a reliable statistic to evaluate dimensionality in the conditions of this simulation. This statistic had a low tendency to reject the two-dimension model when the generating model has three dimensions and the sample is not large. In practice, the conservative behavior of the SGDDM can be seen as a desirable property, as it provides protection against the extraction of dimensions that are not well represented in the data. More investigation would be needed to take the SGDDM as a general measure to evaluate dimensionality of nominal response models in the Bayesian context. With respect to the discrepancy statistics, their real advantage is that they avoid resampling of posterior predictive data matrices and can be computed much more quickly and easily than the SGDDM. However, these results, preliminary as they are, indicate that these statistics should not be used to evaluate model dimensionality.

## Real data analysis

This section describes an exploratory nominal factor analysis in the Bayesian framework using a data sample in the context of learning styles. The purpose is to illustrate the proposed methods in the context of an investigation with real data.

### Instrument

The data set was adopted from a reduced version of the Kolb's (1985) *Learning Styles Inventory, LSI*, which has been widely used in educational and working contexts. Kolb (1984) claims that people naturally prefer a certain type of learning style. Learning style itself results from the combination of two bipolar dimensions: (1) concrete experience (feeling) vs. abstract conceptualization (thinking), and (2) active experimentation (doing) vs. reflective observation (watching). Four learning styles result from the combination of these two variables: (1) accommodating (feeling and doing), (2) diverging (feeling and watching), (3) converging (thinking and doing), and (4) assimilating (thinking and watching).

The original version of the LSI consists of 12 self-report items with 4 response categories that should be rank ordered by the subjects according to their preferences. Each of the categories is designed to load on one of the poles of the bipolar variables: feeling, watching, thinking, and doing. However, the present study is based on a reduced version of the LSI to facilitate the task to the individuals. The reduced version contains four items and is shown in Table 8. Each item contains an incomplete sentence that must be completed with one of the four response categories. The items have a multiple-choice format; the task of the subject consists of selecting the category that better represents his/her preferences.

**Table 8:** Reduced version of the Kolb's Learning Style Inventory.

ITEM 1. I learn best when…
- I rely on my feelings to guide me
- I observe the situation
- I set priorities
- I try out different ways of doing it
ITEM 2. I learn…
- feeling
- watching
- thinking
- doing
ITEM 3. When I learn…
- I like to deal with my feelings
- I like to watch and listen
- I like to think about ideas
- I like to be doing things
ITEM 4. I learn best from…
- personal relationships
- observation
- rational theories
- a chance to try out and practice

### Sample

Subjects were 448 students of the Universidad Católica del Norte (Chile). All the subjects were first-year graduate students: 38% of Psychology, 37% of Engineering, 13% of Architecture, 8% of Journalism, and 4% of Economics. Males and females were equally represented, and ages ranged from 17 to 37 years (mean 19.13 and standard deviation 1.75). These data were collected as part of a larger study of learning preferences involving several questionnaires; thus, it was important to reduce the number of items administered to each individual and to facilitate the task involved by each item. With four items and four categories each one, the number of different response patterns that can be observed is 4^4^ = 256, and there is less than twice the number of individuals than response patterns. The Bayesian framework is appealing with samples of moderate size like this to stabilize estimates.

### Procedure

The classic parameterization of the MNRM in Equation (2) was selected for the analysis of the LSI because the important information that we want to recover is the relation between the categories and the dimensions. Models were estimated using the prior distributions in Equation (19).

### Results

The results of the study are organized according to the three steps explained in Section Estimation of the Model.

#### Selection of the number of dimensions

Step 1 consists of estimating several models with an increasing number of dimensions and parameterized with simple constraints. Models between one and four dimensions were estimated and the simplest model that fits the data was selected. Table 9 shows the result of the model evaluation statistics. Results of the DIC and the SGDDM concur on supporting the model with two dimensions. The model with five dimensions was not estimated because the model with four dimensions was already rejected in favor of simple models based on these results. The model evaluation statistics based on cross-validation showed a tendency to support models with a high number of dimensions. Both the WAIC and LOO give support to the model with four dimensions. However, we have selected the model with two dimensions for interpretation based on the results by the SGDDM and because our simulation studies show that the WAIC and LOO have a tendency to over-factoring. The values of the convergence statistic, *R*, for the selected model ranged from 1.00 to 1.01, which is indicative of good convergence.

**Table 9:** Model evaluation statistics: posterior predictive checks and discrepancy measures.

	**Number of dimensions**			
	**1**	**2**	**3**	**4**
No. of parameters	11(12)448[1]	21(12)896[2]	30(12)1,344[3]	38(12)1,792[4]
*SGDDM*(X;**ω**)	0.071	0.057	0.057	0.057
*SGDDM*(X^*pred*^; **ω**)	0.055	0.058	0.058	0.057
*p_*post*_*	0.005	0.560	0.559	0.567
DIC	4,291.0	5,612.7	8,669.7	13,097.4
WAIC	3,791.8	3,504.1	3,423.4	3,250.2
elpd*_*waic*_*	−1895.9	−1752.1	−1711.7	−1625.1
p*_*waic*_*	301.8	499.0	581.9	638.6
LOO	3,867.0	3,669.6	3,654.1	3,591.1
elpd*_*loo*_*	−1933.5	−1834.8	−1827.0	−1797.1
p*_*loo*_*	339.4	581.8	697.2	810.6

*The number of estimated parameters has the format: number of a (number of d) number of θÂă[number of σ_j_]. p_waic_ and p_loo_ are estimations of the effective number of parameters. elpd_loo_ is the expected log predictive density*.

**Figure 2:**
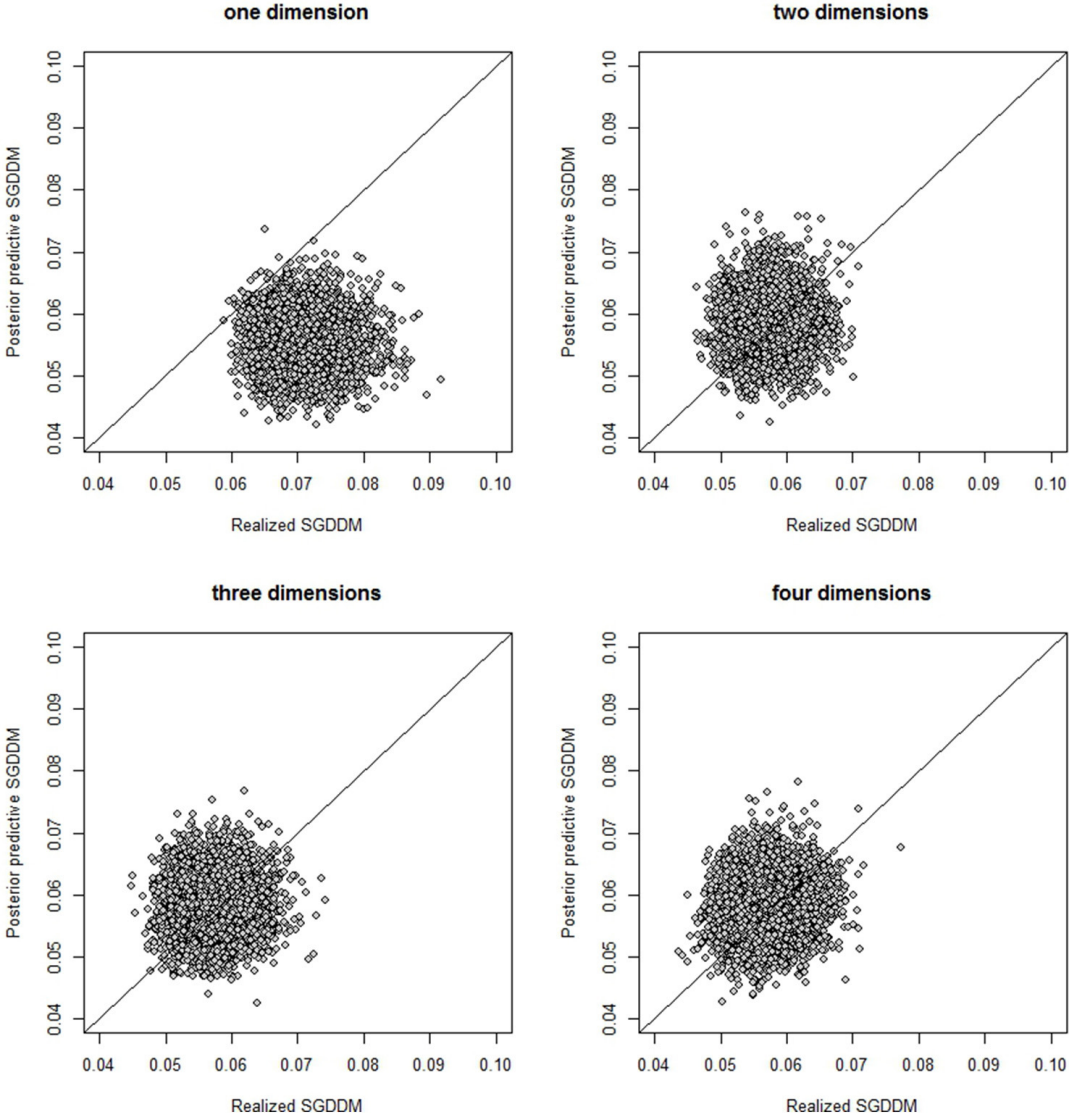
Scatterplot of the realized and posterior predictive values of the SGDDM for the models with one to four dimensions.

The visual inspection of the dispersion plot of *SGDDM*(**X**; **ω**) and *SGDDM*(**X**^*pred*^; **ω**) for the models with one and two dimensions clearly shows that two dimensions are necessary to represent these data. The dispersion plots can be seen in Figure 2. The horizontal axis represents *SGDDM*(**X**; **ω**) and *SGDDM*(**X**^*pred*^; **ω**) is in the vertical axes. The *p*_*post*_ associated to SGDDM in Table 9 is the proportion of points in the figure that fall above the bisection line.

Estimated parameters under simple constraints appear in Table 10. The table shows the pattern of zeros and ones that have to be fixed in the slopes to fix dimension orientation and avoid rotational indeterminacy during estimation, as described in Section Rotation of Slopes. The parameters of category 4 have been set to zero to implement simple constraints.

**Table 10:** Parameter estimates for the two-dimension model under simple constraints.

**Item**	**Category**	**Intercept**	**Dimension 1**	**Dimension 2**
1	1	3.41 (0.49)	**1**	**0**
	2	2.38 (0.50)	0.82 (0.09)	**1**
	3	1.51 (0.49)	0.35 (0.11)	0.52 (0.31)
	4	**0**	**0**	**0**
2	1	5.24 (1.00)	1.77 (0.05)	−1.55 (1.07)
	2	1.77 (0.96)	0.89 (0.37)	0.88 (0.83)
	3	−0.70 (1.31)	0.53 (0.35)	3.89 (1.26)
	4	**0**	**0**	**0**
3	1	2.91 (0.54)	1.57 (0.44)	−1.63 (1.10)
	2	2.42 (1.50)	0.96 (0.27)	0.80 (0.76)
	3	0.61 (0.70)	0.34 (0.24)	3.27 (1.10)
	4	**0**	**0**	**0**
4	1	1.22 (0.43)	0.75 (0.24)	−0.11 (0.71)
	2	0.24 (0.57)	1.05 (0.54)	4.19 (1.32)
	3	0.57 (0.41)	0.55 (0.23)	2.67 (0.90)
	4	**0**	**0**	**0**

*Parameters in boldface are fixed by design. Standard errors appear in brackets. The estimated standard deviation of dimensions are σ_1_ = 2.65 (1.80) and σ_2_ = 0.76 (0.51). The slope for the categories 1 and 2 of item 1 has been set to 1 to fix the orientation of the dimension*.

#### Transformation of parameterization

Simple constraints are not useful for interpreting the LSI questionnaire because category 4 is not a reference category but a substantive one. The information about the relation between category 4 and the dimensions is lost if their parameters are set to zero to resolve the mathematical indeterminacies of the probabilistic model. Simple constraints were transformed to deviation constraints to obtain a more meaningful parameterization in which all categories can have nonzero parameters. The result is on the left part of Table 11. The interpretation of simple constraints has to take into account that the sign of the slopes must change across categories for the sum of the slopes to be zero. Thus, some categories have a positive relation with the dimensions whereas this relation is negative for other categories, which typically results in a bipolar interpretation for the dimensions. Table 11 shows that the slopes in the second dimension have a larger magnitude than in the first one.

**Table 11:** Transformed slopes for the two-dimension model.

		**Deviation constraints**		**Rotated slopes**	
**Item**	**Category**	**Dimension 1**	**Dimension 2**	**Dimension 1**	**Dimension 2**
1	1	0.46	−0.38	0.50	0.32
	2	0.27	0.62	−0.50	0.45
	3	−0.19	0.14	−0.20	−0.14
	4	−0.54	−0.38	0.20	−0.63
2	1	0.98	−2.36	2.54	0.20
	2	0.10	0.07	−0.04	0.11
	3	−0.27	3.09	−3.02	0.69
	4	−0.80	−0.81	0.52	−1.01
3	1	0.85	−2.24	2.40	0.11
	2	0.24	0.19	−0.11	0.29
	3	−0.38	2.66	−2.65	0.46
	4	−0.72	−0.61	0.36	−0.87
4	1	0.16	−1.80	1.76	−0.40
	2	0.46	2.50	−2.24	1.21
	3	−0.04	0.98	−0.95	0.27
	4	−0.59	−1.69	1.43	−1.08

*The parameters labeled Deviation constraints are a transformation of the estimates in Table 4 for the sum of the slopes of each item to be zero. Rotated slopes are obtained from Deviation constraints parameters by an orthogonal rotation*.

#### Rotation of slopes

A visual inspection of parameters under simple constraints reveals that the interpretation could be benefited from a rotation. Rotation was not performed by algorithm procedures such as varimax, oblimin, etc. The reason is that in a simple example like this, with few slopes to rotate, a visual inspection of slopes and the judgment of the data analysis may provide a more meaningful interpretation that these algorithms that are blind to item content. Rotation was performed by a graphical method (Lawley and Maxwell, 1971), by plotting slopes and finding the best rotation angle on a subjective basis. In particular, we performed a clockwise graphical orthogonal rotation by an angle of 72°. The value of the angle is used to compute the transformation matrix, **M**, in Equation (12) using the formula in Lawley and Maxwell (1971, p. 70). The parameters under deviation constraints and the rotated parameters appear in the right part of Table 11.

**Figure 3:**
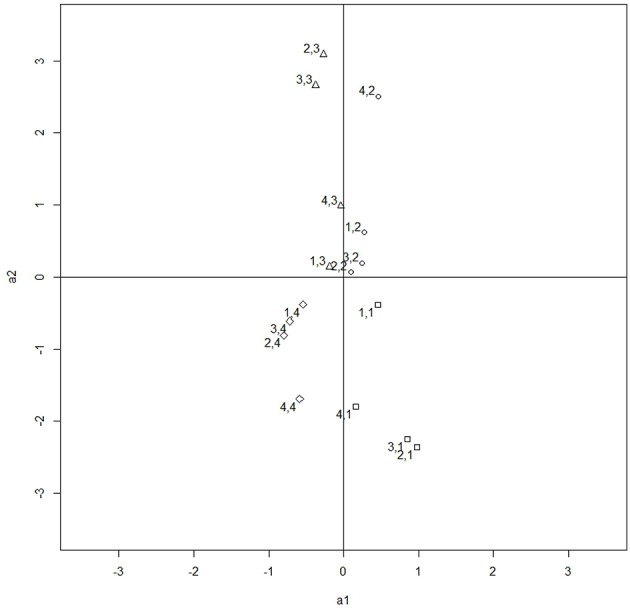
Slopes under deviation constraints. The points are labeled with the number of item and category. For example, the point 4,2 refers to item 4 category 2.

**Figure 4:**
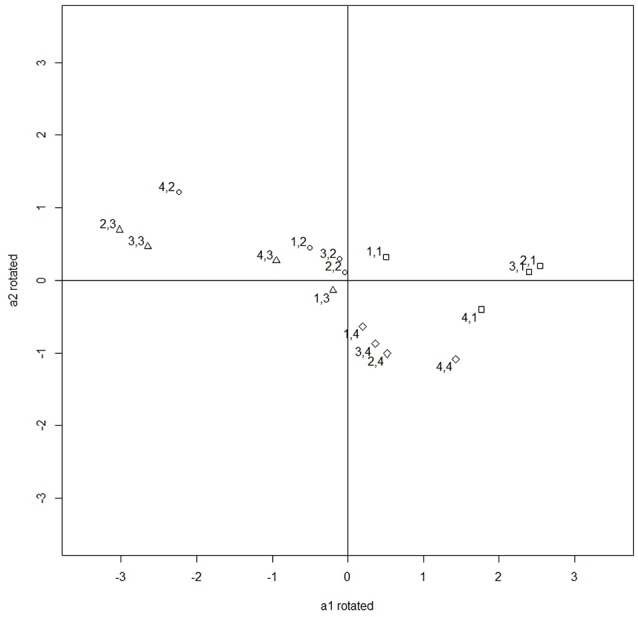
Rotated slopes by an angle of 72°.

Figures 3, 4 illustrate the rotation process. Figure 3 contains the unrotated slopes under deviation constraints and Figure 4 contains the rotated ones. The right part of Table 11 contains the rotated slopes.

The items contain four response categories (feeling, watching, thinking, and doing) that represent the four extremes in the learning model by Kolb (1984). Two bipolar dimensions result from the combination of these extremes. According to Kolb (1981, p. 236):

“The first dimension represents the concrete experiencing of events, at one end, and abstract conceptualization at the other. The other dimension has active experimentation at one extreme and reflective observation at the other.”

In our questionnaire, categories 1 and 3 (feeling and thinking) were designed to represent the two extremes of the first bipolar dimension, whereas categories 2 and 4 (watching and doing) are the two extreme of the second dimension.

The rotated slopes found in the data analysis are in concordance with the theoretical foundation of the questionnaire. The first category of the four items has a positive slope in Dimension 1, and the slope is negative for the third category. Therefore, the probability of category 1 is high when the location of the individual in Dimension 1 is high, and those individuals who are low in Dimension 1 will have a high probability of selecting category 3. Based on these results, Dimension 1 can be interpreted as a bipolar dimension with two extremes: feeling and thinking respectively, which is recognized as the first bipolar dimension in the theoretical model by Kolb.

Similarly, the second category of the four items has a positive rotated slope in Dimension 2 whereas category four has a negative rotated slope. Because the slope indicates the relation of the probability of the category with the dimension, the probability of category two increases with the dimension and the probability of category four increases when the dimension decreases. Thus, the second dimension found in our data is recognized as the second bipolar dimension by Kolb. However, the slopes in the second dimension have a smaller magnitude than those in the first dimension and thus the questionnaire provides less precise measurements in the second dimension.

In conclusion, the two theoretical dimensions postulated by Kolb emerged in our data, which constitutes support for this theoretical model. However, Dimension 1 seems more prominent according to the magnitude of the slopes, and an enlarged version of the questionnaire should be considered to obtain precise estimates in the two dimensions.

## Final remarks

This article described Bayesian methods for evaluating the latent dimensionality of the MNRM, a simulation study, and an example with real data. The initial motivation for moving the inference for the MNRM to the Bayesian context was to alleviate the estimation problems originated by the complex parameterization. However, the drawback of leaving the frequentist framework is the loss of the chi-square and other measures of model fit. For these reasons, it was necessary to define an evaluate Bayesian measures of model adequacy.

The main focus of the article is on dimensionality assessment for the MNRM in the Bayesian context, in particular on the use of the SGDDM for the evaluation of dimensionality. An extension of the SGDDM to the nominal model is introduced and evaluated in a simulation study. Results reveal that the SGDDM is a useful statistic to evaluate dimensionality of the MNRM. This statistic was perhaps a little conservative in small samples, as it showed some tendency to under-factoring. However, this is not necessarily a drawback of the SGDDM because estimates tend to be unstable in small samples. The Bayesian methods implicitly take into account the imprecision of the estimates, and tend to avoid the extraction of those dimension that have a weak empirical support.

The SGDDM was compared in the simulations to three discrepancy measures (DIC, WAIC, and LOO). The discrepancy measures have computational advantages, as they do not require resampling. However, in the conditions of the present investigation they have little utility. The DIC has a strong tendency to under-factoring. The WAIC and LOO were more useful; WAIC was more liberal than LOO and exhibited a preference for models with more dimension, falling on the side of over-factoring in some cases. Thus, the LOO seems the most promising discrepancy measure but its performance is still far from those of SGDDM. All in all, resampling data and computing the SGDDM seems to be the most reliable method for dimensionality assessment in the Bayesian context.

The present investigation can be expanded in several ways. Regarding the discrepancy statistics the most important open problem is the identification of those conditions where these statistics provide valuable information in conjunction with the MRNM. That would be a valuable contribution because discrepancy statistics avoid resampling and are much more computationally cheaper than SGDDM. Vehtari et al. (2016) pointed out that discrepancy statistics can fail with weak priors and sparse data, which is unfortunate because item response models are typically applied to sparse data. Based on this observation, the search of appropriate conditions could start with large sample sizes and/or highly informative prior distributions.

Although the SGDDM is a promising approach to evaluate model dimensionality, it has been tested in a limited number of conditions in the simulation study. The generalization of the present results to other conditions and instruments needs to be further investigated. Levy et al. (2015) have pointed out that the latent structure of the set of items could have an impact on the sensitiveness of the SGDDM. For example when the items are organized in testlets there are testlet-specific dimensions that have zero loadings on the items of the other testlets. Dimensions that affect only to a small number of items could have a small effect on the dimensionality of the complete test and may be hard to detect. One second case in which the performance of the SGDDM shall be investigated is in the presence of weak dimensions that have an effect on all the items but with small slopes (Ximénez, 2006, 2015). In our simulations all the dimensions had high slopes on all the items, which may explain the sensitiveness of the SGDD in detecting statistical association between items. However, additional simulations are needed to investigate if the SGDDM is able to detect dimensions with milder effect in the context of the MNRM.

The simulation study confirmed that prior distributions may help to avoid the problem of high standard errors associated with item parameters. Our analysis revealed that a normal prior is appropriate for the purposes of stabilizing estimates. However, the prior distribution has to be chosen carefully. A too concentrated prior may introduce a bias in the estimated parameters and, on the other hand, a vague prior may lead to problems of convergence and high standard errors (Sheng, 2010). Recently Natesan et al. (2016) investigated the effect of three types of priors (matched, standard vague and hierarchical priors) in Bayesian estimation of dichotomous item response models and recommended the use of hierarchical priors. One line of future research is the investigation of the biases that could be introduced in the MNRM by the use of fixed hyper-parameters and the advantages of hierarchical priors.

One second line of future research is the determination of the minimum number of simulated samples for a simulation study like this is. MCMC simulation is a computationally intensive method and estimation is typically much slower than maximum-likelihood. For these reasons simulation studies tend to use a limited amount of samples. However, a systematic investigation on the minimum number of samples, following the indications by Koehler et al. (2009), would constitute valuable guidelines for researches in the Bayesian item response modeling area.

## Author contributions

The contribution of JR consists of defining the Bayesian estimation and model evaluation procedures for the multidimensional nominal response using Bayesian procedures. JR is also responsible of writing the computer codes in the R and Stan languages and running the simulation study included in the last section of the article. The contribution of CX has focused on the real data analysis section of the article, which describes an application of the exploratory nominal factor analysis in the context of learning styles. This includes the data collection and the analyses of the results. Both authors, JR and CX, have collaborated in writing the paper.

### Conflict of interest statement

The authors declare that the research was conducted in the absence of any commercial or financial relationships that could be construed as a potential conflict of interest.
